# The TRPP2-dependent channel of renal primary cilia also requires TRPM3

**DOI:** 10.1371/journal.pone.0214053

**Published:** 2019-03-18

**Authors:** Steven J. Kleene, Brian J. Siroky, Julio A. Landero-Figueroa, Bradley P. Dixon, Nolan W. Pachciarz, Lu Lu, Nancy K. Kleene

**Affiliations:** 1 Department of Pharmacology and Systems Physiology, University of Cincinnati, Cincinnati, Ohio, United States of America; 2 Division of Nephrology and Hypertension, Cincinnati Children's Hospital Medical Center, Cincinnati, Ohio, United States of America; 3 Department of Chemistry, University of Cincinnati, Cincinnati, Ohio, United States of America; 4 Renal Section, Department of Pediatrics, University of Colorado School of Medicine, Aurora, Colorado, United States of America; Indiana University School of Medicine, UNITED STATES

## Abstract

Primary cilia of renal epithelial cells express several members of the transient receptor potential (TRP) class of cation-conducting channel, including TRPC1, TRPM3, TRPM4, TRPP2, and TRPV4. Some cases of autosomal dominant polycystic kidney disease (ADPKD) are caused by defects in TRPP2 (also called polycystin-2, PC2, or PKD2). A large-conductance, TRPP2-dependent channel in renal cilia has been well described, but it is not known whether this channel includes any other protein subunits. To study this question, we investigated the pharmacology of the TRPP2-dependent channel through electrical recordings from the cilia of mIMCD-3 cells, a murine cell line of renal epithelial origin. The pharmacology was found to match that of TRPM3 channels. The ciliary TRPP2-dependent channel is known to be activated by depolarization and by increasing cytoplasmic Ca^2+^. This activation was greatly enhanced by external pregnenolone sulfate, an agonist of TRPM3 channels. Pregnenolone sulfate did not change the single-channel current-voltage relation. The channels were effectively blocked by isosakuranetin, a specific inhibitor of TRPM3 channels. Both pregnenolone sulfate and isosakuranetin were effective at concentrations as low as 1 μM. Knocking out TRPM3 by CRISPR/Cas9 genome editing eliminated the ciliary channel. Thus the channel is both TRPM3-dependent and TRPP2-dependent, suggesting that it may include both types of subunit. Knocking out TRPM3 did not change the level of TRPP2 protein in the cilia, so it is unlikely that the absence of functional ciliary channels results from a failure of trafficking.

## Introduction

Most cells in the body possess a single primary cilium. Signals generated by primary cilia play critical roles in human health. Defects in primary cilia are implicated in kidney disease, cancer, cognitive impairment, and obesity [1–4]. While the devastating consequences of ciliary pathologies are clear, the mechanisms of signaling by primary cilia are less well understood. An important functional focus has been identified: The primary cilium is specialized for Ca^2+^ signaling [5,6]. Demonstration of this was advanced by the development of methods for recording electrical signals [7,8] and intraciliary Ca^2+^ changes [5,6,9,10] in the native cilia.

Using these and other methods, several Ca^2+^-conducting ion channels have now been identified in the membranes of various primary cilia [11,12]. These channels include TRPC1 [13], TRPM3 [14–16], TRPP2 [17–20], TRPP3 (also called PKD2-L1 [5,8]), TRPV4 [15,21], and L-type Ca^2+^ channels [6]. Some cilia express several of these channel types, and it is an open question how the channels function together to influence ciliary Ca^2+^ signals. Investigations are complicated by the possibility of heteromultimeric channels. Channels of the transient receptor potential (TRP) family typically contain four protein subunits, but the subunits are not necessarily identical. TRPP2, for example, can form heteromeric channels that also include TRPC1 [13] or TRPV4 [21,22] subunits.

In primary cilia of renal epithelial cells, a channel that requires TRPP2 (also called polycystin-2, PC2, or PKD2; the product of the *Pkd2* gene) is now well described [19,20]. Defects in TRPP2 cause some cases of autosomal dominant polycystic kidney disease [23]. It is not known whether the ciliary TRPP2 channel includes other protein subunits. In addition to TRPP2, kidney cells express several other members of the TRP family of cationic channels, including TRPM3 [15,24,25]. In testing reagents that modulate TRPM3 activity, we unexpectedly found that they strongly influence the ciliary TRPP2-dependent channel. We further found that knocking out TRPM3 eliminates the ciliary TRPP2-dependent channel. That channel, in other words, requires expression of both TRPM3 and TRPP2. The results suggest that the ciliary channel may be a heteromultimer that includes both TRPM3 and TRPP2 subunits. We identify an activator of this channel, pregnenolone sulfate, and discuss the possibility that it may be beneficial in treating cystic kidney disease.

## Materials and methods

### Electrical recording

**Fig 1:**
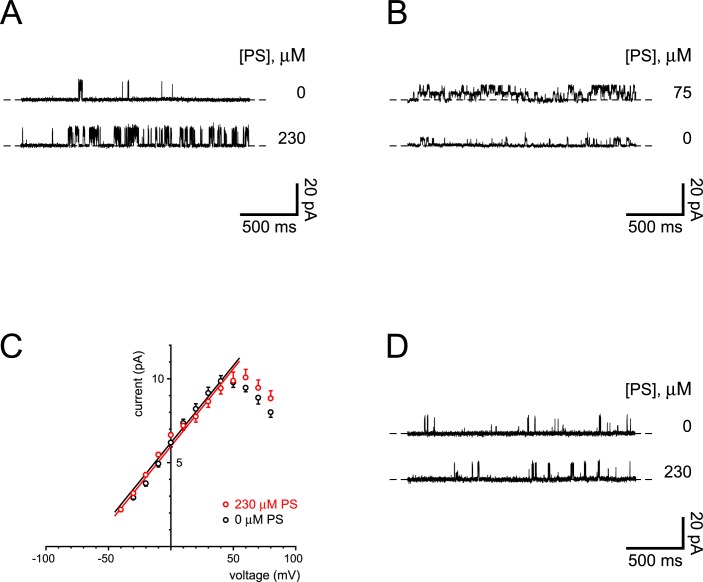
Activation of ciliary channels by pregnenolone sulfate. (A) The top recording shows the activity of a single ciliary channel observed while recording in the standard solutions (with 0.1 μM cytoplasmic free Ca^2+^). The bottom recording shows increased channel activity in the same cilium 5 min after perfusion of the pipette with standard external solution to which 230 μM pregnenolone sulfate had been added. The holding potential was +60 mV for both recordings. The dashed lines indicate the current level when the channel was closed. (B) The top recording shows the activity of two ciliary channels observed with the standard pipette (external) solution to which 75 μM pregnenolone sulfate had been added. The bottom recording shows decreased channel activity in the same cilium 4 min after perfusion of the pipette with the standard external solution, which lacks pregnenolone sulfate. For both recordings, free Ca^2+^ was 3 μM and the holding potential was −10 mV. (C) Single-channel current-voltage relation measured in standard external solution without (black) or with (red) 230 μM pregnenolone sulfate. For all recordings, cytoplasmic free Ca^2+^ was either 0.1 or 3 μM. Each point shown is the mean of measurements in 3 to 5 cilia (without pregnenolone sulfate) or 7 to 10 cilia (with pregnenolone sulfate). Each straight line is fit to the linear portion of its relation (−40 to +50 mV; *R*^2^ 0.972, *P* < 0.001 without pregnenolone sulfate; *R*^2^ 0.992, *P* < 0.001 with pregnenolone sulfate). (D) The top recording shows the activity of a single ciliary channel observed while recording in the standard solutions. The bottom recording shows channel activity in the same cilium 5 min after replacing the cytoplasmic solution with one containing 230 μM pregnenolone sulfate. Both cytoplasmic solutions contained 0.1 μM free Ca^2+^. The holding potential was +60 mV for both recordings.

Electrical recordings were made from primary cilia of mIMCD-3 cells as described previously [7]. In short, mIMCD-3 cells (murine epithelial cells from the renal inner medullary collecting duct, CRL-2123, American Type Culture Collection, Manassas, Virginia, USA [26]) were cultured on beads that were free to move in the recording chamber. Suction was applied to a recording pipette so that a single primary cilium entered the pipette. If a resistance of at least 1 GΩ formed between the membrane and the pipette, the cilium was excised from the cell. This left the cilium inside the recording pipette in the inside-out configuration. The pipette containing the cilium could then be transferred among different solutions that bathed the cytoplasmic face of the membrane. (A photograph [7] and a schematic diagram [19] appear elsewhere.) In one set of experiments (Fig 1, parts A and B), the external solution was replaced by perfusing the pipette as described previously [27,28].

During recording, the beads coated with cells were stored in a standard external solution containing (in mM) NaCl 140, KCl 5, CaCl_2_ 2, MgCl_2_ 2, sodium pyruvate 2, HEPES 5, and D-glucose 9.4, adjusted to pH 7.4 with NaOH. The recording pipettes also contained this solution except as noted. In some experiments pregnenolone sulfate was added to the external solution as noted. The standard cytoplasmic solution contained (in mM) KCl 140, NaCl 5, CaCl_2_ 0.7, MgCl_2_ 2, HEPES 5, BAPTA 2, and D-glucose 5, adjusted to pH 7.4 with KOH. This solution contained 0.1 μM free Ca^2+^. To make a cytoplasmic solution with 3 μM free Ca^2+^, BAPTA was replaced with dibromoBAPTA, and the total CaCl_2_ was increased to 1.4 mM. In buffered solutions, concentrations of free Ca^2+^ were estimated by the method of Bers [29] as described previously [30]. Pregnenolone sulfate or isosakuranetin was added to the cytoplasmic solution as noted.

All recordings were done under voltage clamp at room temperature (24°C). Equipment, software, and technical details, including corrections for liquid junction potentials, were as previously described [19]. During acquisition, currents were low-pass filtered at 2 kHz and digitized at 5 kHz. Total mean channel current was measured as the mean current minus the current attributed to leak channels. The latter was determined from an amplitude histogram. In each cilium reported, the number of large-conductance channels was between 1 and 5. Channel open probabilities were determined from amplitude histograms and are reported only when the number of channels in the membrane was unambiguous. The presence or absence of active TRPP2 channels was assessed with the cilium or patch exposed to 3 μM cytoplasmic Ca^2+^ and the voltage clamped to +40 mV. If no large-conductance channels were seen to open within 2 min, active TRPP2 channels were judged to be absent. These cilia have a second Ca^2+^-activated channel, TRPM4, but it is not activated by the concentrations of cytoplasmic Ca^2+^ used in this study (0.1 or 3 μM). The half-maximal effect of Ca^2+^ on ciliary TRPM4 occurs at 646 μM at +100 mV and 1166 μM at −100 mV [31].

### Concentration of pregnenolone sulfate

For the electrophysiological studies, pregnenolone sulfate was prepared at nominal concentrations up to 300 μM in standard external solution. The 300 μM stock was sonicated but did not appear by visual inspection to be completely dissolved. In order to determine the maximum stable concentration of pregnenolone sulfate in the external solution, aliquots of pregnenolone sulfate in DMSO were diluted in external solution to final concentrations of 50, 200, 300, and 500 μM in duplicate, sonicated in a sonication bath (Thermo Fisher Scientific, Waltham, Massachusetts, USA) for 1 min, and left at room temperature (25°C) for 10, 30, or 60 min. The samples were then centrifuged at 10,000 × *g* for 2 min, and for each an aliquot of the supernatant was diluted 20× with doubly distilled water for analysis. Given the simple nature of the samples, HPLC-UV/Vis was used to determine the pregnenolone sulfate concentrations by adapting the method of Sánchez-Guijo et al. [32]. In short, 5 μL of each sample was injected into an HPLC system consisting of an Agilent 1100 HPLC equipped with a membrane solvent degasser, a binary pump, a thermostatted auto sampler, thermostatted column compartment, and diode array UV/Vis flow-cell detector with a 10-mm optical path. An Agilent Zorbax Phenyl column (4.6 × 250 mm, 5 μm particle size) equipped with a C-18 pre-column cartridge was used with the mobile phases A (10 mM ammonium acetate, pH 7, in 85% water and 15% acetonitrile, *v*/*v*) and B (70% methanol and 30% acetonitrile). The total flow rate was 1 mL min^−1^ with a gradient as follows: 0 min, 80% A / 20% B; 20 min, 30% A / 70% B; 23 min, 1% A / 99% B; 25 min, 1% A / 99% B; 28 min, 80% A / 20% B. An equilibration time of 10 min was used after each injection, and the absorbance at 215 nm (A_215_) was used for quantification based on the peak height.

A set of calibration standards in doubly distilled water was prepared at 50, 100, 150, 200, 250, 300, 350, and 500 μM. This water-based calibration was used to quantify the relation between pregnenolone sulfate concentration and A_215_ (S1 Fig). A good agreement with the water-based calibration was observed for the concentrations of 50 μM and 200 μM in external solution, while 300 μM or above showed little increase in pregnenolone sulfate concentration. For the experiments shown in Results, the highest nominal concentration of pregnenolone sulfate was 300 μM. The true concentration in that case averaged 232 ± 9 μM (S1 Fig) and is reported in Results as 230 μM.

### Cell lines

The development and characterization of the TRPM3-knockout and TRPP2-knockout mIMCD-3 clonal lines have been described previously [15,19]. Since the mIMCD-3 line is nearly triploid [33], we note that we sequenced the DNA at the mutation site in the TRPM3-knockout and TRPP2-knockout clones and never saw the wild-type sequence; we only detected mutated sequences.

### Western blotting

Wild-type mIMCD-3 cells, two mIMCD-3 clones with TRPM3 knocked out, and one with TRPP2 knocked out were grown for 7 to 10 d past confluence on plastic, tissue-culture-treated Petri dishes in DMEM/F12 medium (10-092-CV, Thermo Fisher Scientific) with 10% fetal bovine serum (97068–085, VWR, Radnor, PA) and 1% penicillin/streptomycin (30-002-Cl, Thermo Fisher Scientific). Cells were rinsed with cold phosphate buffered saline (PBS) three times. PBS contained (in mM) KCl 2.7, KH_2_PO_4_ 1.5, Na_2_HPO_4_ 8.1, NaCl 140, pH 7.4. The cells were scraped from the dish and transferred to a centrifuge tube with a small amount of PBS. Cells were pelleted at 190 × *g* / 4°C for 5 min. After removing the supernatant, the cells were resuspended in low ionic strength lysis buffer (10 mM Tris-HCl, 0.5 mM MgCl_2_) that contained protease inhibitors (Halt Protease inhibitor cocktail, #87785, Thermo Fisher Scientific) at 10 μL per 1 mL of buffer. The cells were incubated on ice for 10 min and then homogenized with a glass homogenizer. The lysates were centrifuged at at 2,000 × *g* / 4°C for 5 min. Membrane protein was isolated by centrifugation of the supernatant at 110,000 × *g* / 4°C for 90 min. The resulting pellet was solubilized in membrane buffer (50 mM Tris-HCl, 150 mM NaCl, 1 mM EDTA, 0.05% sodium deoxycholate, 0.1% sodium dodecyl sulfate, 1% Triton X-100) and protease inhibitors with sonication (15 s with a Cell Disruptor Sonicator (setting 4 on model W-220F, Qsonica, Newtown, CT)). The membrane protein was centrifuged at 12,000 × *g* / 4°C for 5 min. Protein concentration of the supernatant was determined with a BCA assay (Thermo Fisher Scientific). Equal amounts (6 μg) of reduced, denatured protein were loaded into wells of a NuPAGE Novex 3–8% Tris-acetate gel (Invitrogen/Thermo Fisher Scientific) and subjected to electrophoresis with sodium dodecyl sulfate at 150 V per Invitrogen’s protocol. Proteins were transferred to a PVDF membrane (Immobilon-FL, Fisher Thermo Scientific) for 1 h at 30 V. After air drying, the membrane was labeled with Revert total protein stain per instructions (LI-COR BioSciences, Lincoln, NE), air dried, scanned on an Odyssey CLx Infrared Imaging System (LI-COR BioSciences), and destained. The membrane was blocked in 50% PBS/50% Odyssey blocking buffer (LI-COR BioSciences) and incubated with an antibody to TRPP2 (1/750 dilution, sc-25749, Santa Cruz Biotechnology, Dallas, Texas, USA) overnight at 4°C per LI-COR instructions. The membrane was incubated with goat anti-rabbit IgG conjugated to Alexa Fluor 790 (1/15,000, A11369, Molecular Probes/Thermo Fisher Scientific). The membrane was air dried and scanned. Empiria Studio software (version 1.0.1.53, LI-COR Biosciences) was used for analysis; it includes a proprietary method of background subtraction. Six cell passages of the four cell lines were tested with one blot per passage and two lanes per cell line on each blot. The fluorescent signal of each lane’s TRPP2 band was normalized by dividing by a lane normalization factor, which was the Revert total protein stain’s fluorescent signal for that entire lane divided by the Revert total protein stain’s fluorescent signal for the brightest lane on the blot. To obtain the normalized TRPP2 labeling value, each lane's ratio of TRPP2 signal/lane normalization factor was divided by the average of the TRPP2 signal/lane normalization factor for the two lanes containing the wild-type samples for that blot. We confirmed that 6 μg/lane was in the linear part of the μg/lane vs. signal curve for Revert total protein stain and TRPP2 signal per instructions (LI-COR BioSciences).

### Quantification of ciliary TRPP2 immunolabeling

Wild-type mIMCD-3 cells, two mIMCD-3 clones with TRPM3 knocked out, and one with TRPP2 knocked out were grown for 4 to 8 d past confluence on #1.5 coverslips in DMEM/F12 medium (10-092-CV, Thermo Fisher Scientific) with 10% fetal bovine serum (97068–085, VWR or 16000–044, Thermo Fisher Scientific) and 1% penicillin/streptomycin (30-002-Cl, Thermo Fisher Scientific) They were fixed with paraformaldehyde with a pH shift, treated with 1% sodium dodecyl sulfate for antigen retrieval, and blocked in a donkey serum-containing buffer as described previously [19]. They were sequentially immunolabeled with a rabbit polyclonal anti- TRPP2 antibody (1/250, sc-25749, Santa Cruz Biotechnology) overnight and a mouse monoclonal antibody against ARL13B (ADP-ribosylation factor-like protein 13B, a ciliary marker [34], 1/1000, N295B/66, Antibodies Incorporated, Davis, California, USA) for 1 h. Antibodies were diluted in 1% BSA and 0.02% sodium azide in PBS. The cells were incubated with Alexa Fluor-conjugated secondary antibodies (donkey anti-mouse Alexa Fluor plus 488 (A32766) and donkey anti-rabbit Alexa Fluor 594 plus (A32754), Thermo Fisher Scientific). They were mounted using Prolong Diamond mounting medium (Thermo Fisher Scientific) and allowed to cure for a minimum of 5 d. Oversampled (100× oil objective with 1.45 numerical aperture, 0.06 μm XY resolution, 0.1 μm Z step, 0.5 Airy unit pinhole, 1.1 μs pixel dwell, 2× integration) three-dimensional stacks of images were acquired on a confocal microscope (A1R, Nikon, Melville, New York, USA). The image stacks were coded to blind the investigator and then deconvolved (Lucy-Richardson, 20 iterations, NIS-Elements software, Nikon) to reassign out-of-focus data. The coded, deconvolved stacks were analyzed with Imaris software (Bitplane, Zürich, Switzerland). Here, we used the ARL13B immunolabeling to define each ciliary volume and then determined the median intensity of TRPP2 labeling per ciliary volume (S3 Fig). The same intensity was used as a threshold for ARL13B immunolabeling for all images. Cilia that were not fully captured in the stack were excluded from analysis. Sometimes cilia were not detected as a single volume, so the number of volumes is greater than the number of cilia. Volumes of surface area less than 0.02 μm^2^ were excluded. On occasion, spherical structures that were similar in shape and size to background, cytoplasmic ARL13B labeling reached threshold and then were excluded manually. (See S3 Fig, cilium #1, row 3, column 2 for an example of background, cytoplasmic ARL13B labeling.) All volumes with surface areas greater than 0.02 μm^2^ that were near other ciliary volumes were included because cilia sometimes formed multiple volumes. Four separate passages of all four cell lines were analyzed. Six fields per cell line and passage were examined to generate 96 image stacks.

### Materials

Pregnenolone sulfate (sodium salt) and BAPTA were purchased from Sigma-Aldrich (St. Louis, Missouri, USA); dibromoBAPTA from Molecular Probes / Thermo Fisher Scientific; and isosakuranetin from Extrasynthese (Genay, Rhône, France). Pregnenolone sulfate and isosakuranetin were diluted from stock solutions in DMSO (100 mM for pregnenolone sulfate and 10 mM for isosakuranetin).

### Statistical analyses

**Fig 2:**
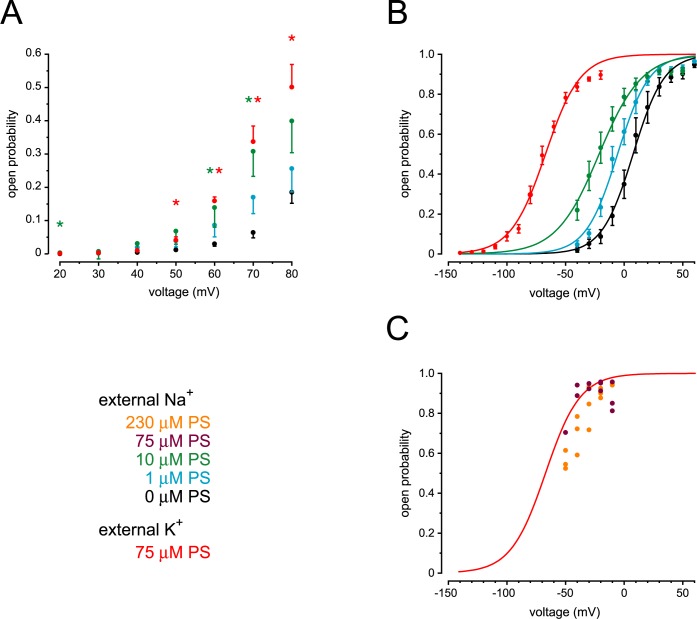
Dependence of channel activation on voltage and pregnenolone sulfate concentration. (A) Channel open probability was measured as a function of membrane potential. Cytoplasmic solutions contained 0.1 μM free Ca^2+^. The pipette contained pregnenolone sulfate (0 μM to 75 μM as indicated) dissolved in an external solution that also contained 0.3% (*w*/*v*) DMSO. Each point shown is the mean of measurements in 4 to 8 cilia. * Significantly different from 0 μM pregnenolone sulfate for the given voltage, *P* < 0.001–0.014, *t*-tests and Mann-Whitney rank sum tests controlled by false discovery rate correction for 21 comparisons. (B) The same procedures as in A but with 3 μM cytoplasmic free Ca^2+^. The external (pipette) solution included pregnenolone sulfate (0 μM to 75 μM as indicated). Each point shown is the mean of measurements in 4 to 11 cilia. The solid smooth curves represent best fits of the open probability-voltage relations to Boltzmann functions as described previously [19]. Constants and strength and significance of fit for the best-fitting Boltzmann functions are given in S1 Table. Representative single-channel recordings are shown in S2 Fig. (C) Responses in standard external solution to which was added 75 μM (purple circles) or 230 μM (orange circles) pregnenolone sulfate. As in (B), cytoplasmic solutions contained 3 μM free Ca^2+^. The red Boltzmann function is copied from (B) (response to 75 μM pregnenolone sulfate with a KCl-based external solution).

Statistical analyses were performed with the critical significance level α = 0.05. Unless otherwise noted, data are presented as mean and standard error (SE) for *n* independent observations. Parametric tests were used when the Shapiro–Wilk test for normality was passed, and nonparametric tests were used when it failed. Student’s *t*-tests reported are two-tailed. When data were fit using linear (Fig 1C) or nonlinear (Fig 2B) regression analysis, the results are expressed as the estimates of fit parameters ± standard error (SE); *R*^2^ is the regression coefficient (strength of the fit) and *P* describes the significance of the fit. CI is the 95% confidence interval.

## Results

The primary cilia of renal epithelial cells express a large-conductance, cationic channel that is absent unless TRPP2 is expressed. The conductance properties of this channel have been described [19,20]. These cilia also express the channel protein TRPM3, but this has only been demonstrated by immunocytochemistry [15]. We examined possible interactions between the TRPM3 and TRPP2 channel subunits by recording transmembrane currents in single cilia excised from mIMCD-3 cells.

In cilia expressing the large-conductance channel, depolarization to +60 mV caused infrequent openings of the channel (Fig 1A, top recording). When the external (pipette) solution was replaced by perfusing the pipette with a solution to which 230 μM pregnenolone sulfate had been added, the open probability of the channel was greatly increased (Fig 1A, bottom recording). Pregnenolone sulfate is an agonist of TRPM3 channels [35,36]. On average, external pregnenolone sulfate increased the mean channel current by a factor of 30 ± 12 (*n* = 6). By contrast, perfusions with a control solution lacking pregnenolone sulfate increased the channel current by a factor of 3.3 ± 0.9 (*n* = 7). The activation by pregnenolone sulfate was significant (*P* = 0.028, *t*-test for independent measures). The activation was reversible. With 75 μM pregnenolone sulfate in the external solution, the channels were very active at −10 mV (Fig 1B, top recording). After replacing the external solution with one lacking pregnenolone sulfate, the fraction of the mean channel current remaining was 0.14 ± 0.08 (Fig 1B, bottom recording; CI 0.22, *n* = 5).

The current-voltage relation of channels activated by 230 μM external pregnenolone sulfate is shown in Fig 1C (red). The single-channel conductance and extrapolated reversal potential closely match those of the TRPP2-dependent channels measured in the absence of pregnenolone sulfate (Fig 1C, black). The mean single-channel conductance (slope) without pregnenolone sulfate is 94 ± 6 pS, and the extrapolated reversal potential (*x*-intercept) is −64 ± 4 mV. The values with pregnenolone sulfate are not significantly different (92 ± 3 pS and −67 ± 2 mV; *P* = 0.78 and *P* = 0.54 respectively, *t*-tests for independent measures). The decrease in conductance at strongly depolarizing potentials (Fig 1C) is characteristic of the TRPP2-dependent channels in the cilia of mIMCD-3 cells [19]. Application of pregnenolone sulfate did not increase the incidence of the channels (Table 1, wild type). In untreated cilia, the TRPP2-dependent channels were apparent in 34% of cilia tested [19]. In cilia exposed to pregnenolone sulfate, channels were observed in 30% of the cilia (Table 1, wild type). These incidences were not significantly different (*P* = 0.32, Fisher’s exact test). The channels were rare in the apical non-ciliary membrane with or without pregnenolone sulfate (Table 1, wild type). The channels are not detectable in the cilia of cells lacking TRPP2 [19]. Addition of pregnenolone sulfate did not reveal any channels in the cilia or apical non-ciliary membrane of cells lacking TRPP2 (Table 1, TRPP2 KO).

**Table 1:** Incidences of TRPP2-dependent channels in apical and ciliary membranes.

membrane	PS	cell line		
		wild type	TRPP2 KO	TRPM3 KO
ciliary	**−**	103 / 304 [19]	0 / 36 [19]	0 / 45
	**+**	77 / 259	0 / 28	ND
apical	**−**	1 / 22 [19]	ND	ND
	**+**	3 / 57	0 / 30	0 / 34

The presence or absence of active TRPP2 channels was assessed with the cilium or apical membrane patch exposed to 3 μM cytoplasmic Ca^2+^ and the voltage clamped to +40 mV. If no large-conductance channels were seen to open within 2 min, active TRPP2 channels were judged to be absent. Where indicated, pregnenolone sulfate was included in the external (pipette) solution at either 75 μM or 230 μM. PS, pregnenolone sulfate; KO, knockout; ND, not determined.

Pregnenolone sulfate was much less effective when applied to the cytoplasmic surface of the cilium (Fig 1D). Cytoplasmic pregnenolone sulfate (230 μM) on average increased the mean channel current by a factor of 3.6 ± 0.7 (*n* = 7). This is a significantly smaller increase than seen with 230 μM external pregnenolone sulfate (*P* = 0.030, *t*-test for independent measures). In both cases cytoplasmic Ca^2+^ was 0.1 μM.

The TRPP2-dependent channel of renal primary cilium is activated by both depolarization and by micromolar levels of cytoplasmic Ca^2+^ [19,20]. When cytoplasmic Ca^2+^ is low (0.1 μM), the channel can be activated by strong depolarization (Fig 2A, black circles). External pregnenolone sulfate significantly increased the channel activity (Fig 2A). Even when the channel was activated by cytoplasmic Ca^2+^, pregnenolone sulfate caused an additional activation. In the presence of 3 μM cytoplasmic Ca^2+^, pregnenolone sulfate at concentrations of 1 μM to 75 μM significantly shifted the potential for half-maximal activation to more negative values (Fig 2B; one-way ANOVA with Holm-Sidak all pairwise comparison; *P* < 0.001 for all comparisons). Pregnenolone sulfate was no more effective at its solubility limit (230 μM, orange circles) than at 75 μM (red curve, Fig 2C).

To determine the relations shown in Fig 2, external solutions were chosen that allowed detection of single-channel events at or near the half-maximally effective voltages. For 0 μM, 1 μM, and 10 μM pregnenolone sulfate, the standard external solution was used, and the channel current reversed at −67 mV. For 75 μM pregnenolone sulfate, an external solution with KCl instead of NaCl was used, and the channel current reversed near 0 mV. The choice of external solution does not account for the high channel activity in 75 μM pregnenolone sulfate. Even in NaCl-based external solution, channel activity was high at voltages where single-channel events were large enough to be measured (Fig 2C, purple circles).

**Fig 3:**
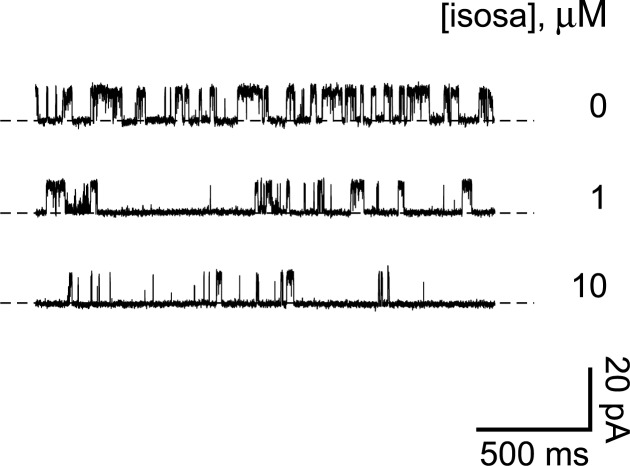
Isosakuranetin reduces the activity of a ciliary channel. The top recording shows the activity of a single ciliary channel in the absence of isosakuranetin. The middle and bottom recordings show reduced channel activity in the same cilium following transfer to solutions containing 1 μM (5 min) or 10 μM (2 min) isosakuranetin, respectively. In all cases cytoplasmic free Ca^2+^ was 3 μM and the holding potential was +30 mV. Isosa, isosakuranetin.

Pregnenolone sulfate activates the TRPP2-dependent channels but is best known as an agonist of TRPM3. For that reason we also tested isosakuranetin, a specific inhibitor of TRPM3 that acts from the cytoplasmic face of the membrane [37]. At +30 mV in the presence of 3 μM cytoplasmic Ca^2+^ (and with no pregnenolone sulfate present), the TRPP2-dependent channel opened frequently (Fig 3, top recording). Addition of cytoplasmic isosakuranetin greatly reduced the open probability of the TRPP2-dependent channel (Fig 3, middle and bottom recordings). The fraction of the mean channel current remaining was 0.41 ± 0.09 with 1 μM isosakuranetin (CI 0.23, *n* = 7) and 0.09 ± 0.04 with 10 μM isosakuranetin (CI 0.09, *n* = 8). Inhibition by 10 μM isosakuranetin was not reversible. After 10 min in solution lacking the inhibitor, the fraction of the original uninhibited current never exceeded 0.09 (*n* = 4).

The large-conductance ciliary channel is not detectable unless TRPP2 is expressed [19,20]. Given the pharmacological similarities between the ciliary TRPP2-dependent channel and TRPM3, we investigated whether the ciliary channel also requires expression of TRPM3. We have previously described an mIMCD-3 cell line in which TRPM3 was knocked out by CRISPR/Cas9 genome editing [15]. In electrical recordings from the primary cilia of cells lacking TRPM3, the large-conductance channel was never seen (*n* = 45, Table 1, TRPM3 KO). This is significantly different from the channel’s incidence in cilia from wild-type cells (34%, Table 1, wild type; *P* < 0.001, Fisher’s exact test). TRPM3-knockout cells also showed no such channels in the apical non-ciliary membrane even in the presence of pregnenolone sulfate (Table 1, TRPM3 KO). Knocking out TRPM3 had no effect on the activity of another ciliary channel, TRPM4 [31]. In 17 cilia tested from TRPM3-knockout cells, 1 mM cytoplasmic Ca^2+^ on average activated a current at +100 mV of 47 ± 7 pA. This is not significantly different from the activity in wild-type cells (50 ± 5 pA, *n* = 88 [31]; *P* = 0.65, Mann-Whitney). As in cilia of wild-type cells, 2 mM cytoplasmic MgATP blocked the TRPM4 current. In cilia lacking TRPM3, the fraction of TRPM4 current remaining after addition of MgATP was 0.34 ± 0.03 (CI 0.07, *n* = 16).

**Fig 4:**
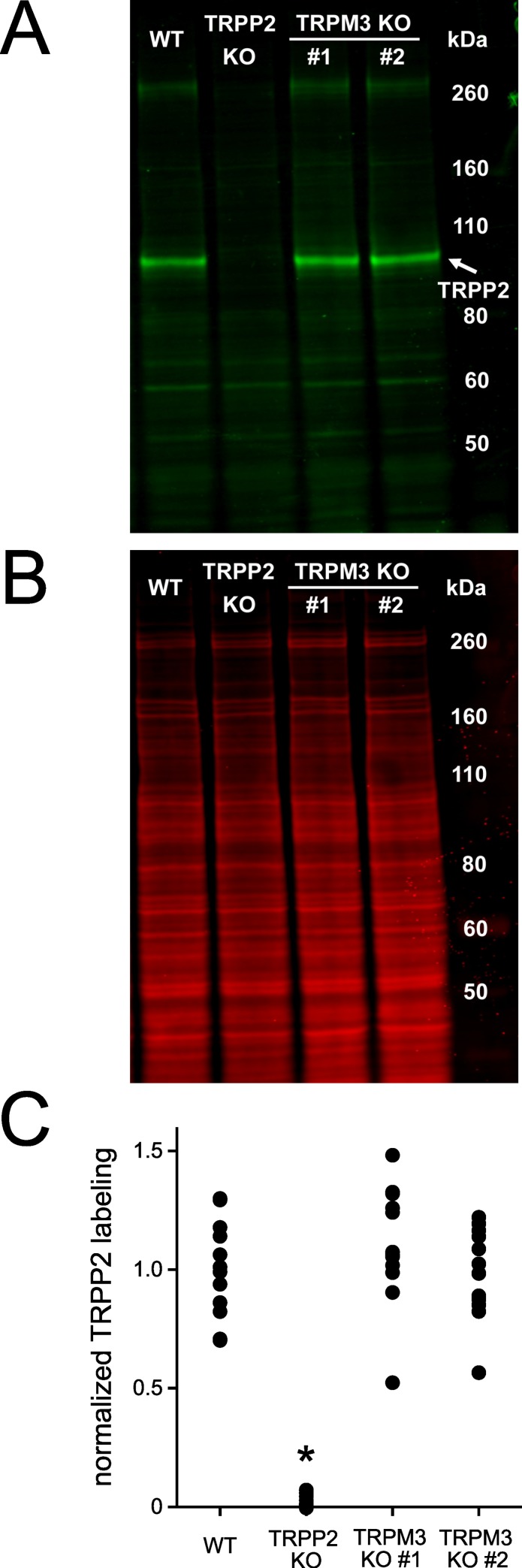
Knockout of TRPM3 does not change TRPP2 membrane protein levels. (A,B) Representative western blot of membrane proteins labeled for TRPP2 (A) or total protein (B). TRPP2 is present in wild-type (WT) and TRPM3-knockout (TRPM3 KO) cell lines but absent from the TRPP2-knockout (TRPP2 KO) cell line. Each blot had a duplicate set of lanes also used for analysis but not shown in the figure. (C) Quantification of TRPP2 labeling. Membrane protein labeling for TRPP2 in two TRPM3 knockout cell lines is not significantly different from that in the wild type (*P* = 0.75 and *P* ≥ 0.99, one-way ANOVA with Tukey's all pairwise comparison). The TRPP2 knockout has less labeling than the other lines (* *P* < 0.001). Six cell passages of the four cell lines were tested with one blot per passage and two lanes per cell line on each blot. The fluorescent signal of each lane’s TRPP2 band was normalized by dividing by a lane normalization factor, which was the Revert total protein stain’s fluorescent signal for that entire lane divided by the Revert total protein stain’s fluorescent signal for the brightest lane on the blot. To obtain the normalized TRPP2 labeling value (*y*-axis), each lane's ratio of TRPP2 signal/lane normalization factor was divided by the average of the TRPP2 signal/lane normalization factor for the two lanes containing the wild-type samples for that blot.

**Fig 5:**
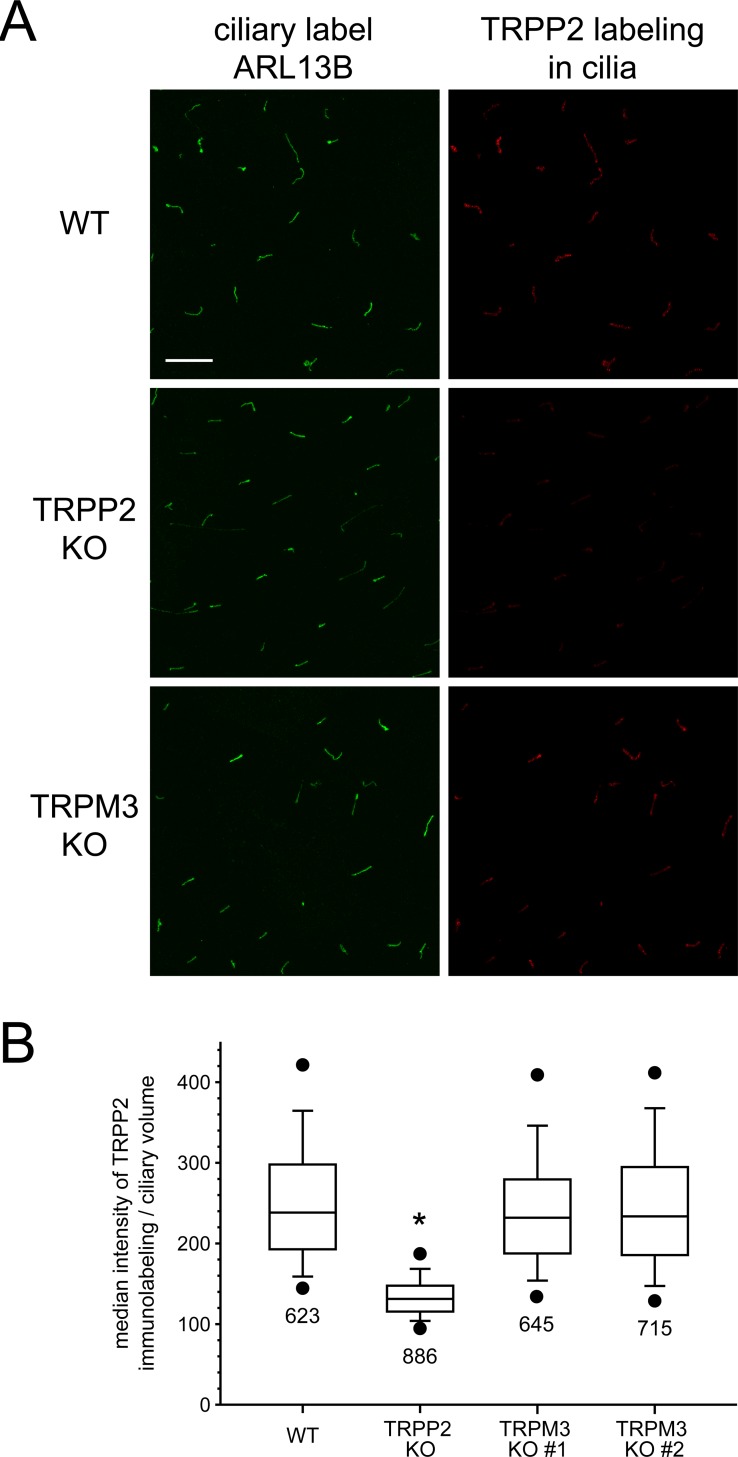
Ciliary TRPP2 immunolabeling is not affected by knocking out TRPM3. (A) For each of the wild-type (WT), TRPP2-knockout (TRPP2 KO), and TRPM3-knockout (TRPM3 KO) cell lines, representative maximum intensity projections through a Z stack of images are shown. The cells were immunolabeled for ARL13B, a ciliary protein [34] (first column). The ARL13B labeling was used to define ciliary volumes in the 3D data set as described in Materials and Methods. The maximum intensity projection for the TRPP2 labeling in those ciliary volumes (second column) shows that knocking out TRPM3 does not alter the TRPP2 ciliary labeling. Knocking out TRPP2 reduces the TRPP2 ciliary fluorescence but non-specific labeling remains. Bar = 10 μm. All images had contrast enhanced in the same manner. A few brightly labeled pixels were saturated so that lightly labeled pixels would be visible while keeping gamma at 1. No pixels were saturated for the quantification of intensity used to make the graph in (B). (B) A box plot of the median intensity of TRPP2 immunolabeling/ciliary volume for each cell line. Knocking out TRPM3 does not alter the TRPP2 ciliary labeling (*P* = 0.62 and *P* ≥ 0.99, Kruskal-Wallis one-way ANOVA on ranks with Dunn’s all pairwise comparison). Knocking out TRPP2 reduces the TRPP2 ciliary fluorescence by about half compared to the other lines (* *P* < 0.001). The remaining fluorescence in the TRPP2 knockout is considered non-specific labeling by the non-affinity purified, polyclonal TRPP2 antibody, which had several non-specific bands in a western blot of whole-cell lysate from these same wild-type and TRPP2-knockout cell lines (see Fig 9A in [19]). The box plot's top, middle, and bottom lines indicate 75th, 50th, and 25th percentile values, respectively. Whiskers indicate 90% and 10% values; circles indicate the 95% and 5% values. The numbers below each box plot indicate the number of ciliary volumes followed. Four cell passages were used for each cell line.

The dependence of functional ciliary TRPP2-dependent channels on TRPM3 expression could suggest that TRPM3 is required for trafficking of TRPP2 to the cilium. Expression of TRPP2 in either of two TRPM3-knockout lines was not significantly changed as judged by western blotting of the membrane protein fraction (Fig 4). As judged by immunocytochemistry, cells lacking TRPM3 were found to retain expression of TRPP2 protein in the cilia (Fig 5).

## Discussion

The primary cilia of renal epithelial cells express a large-conductance cationic channel. This channel is absent if the TRPP2 channel protein is genetically eliminated [19,20]. We now report that, in the renal epithelial cell line mIMCD-3, this same channel also requires expression of a second channel subunit, TRPM3. Furthermore, the channel displays pharmacological properties characteristic of TRPM3 channels [25,38].

In renal primary cilia, both TRPM3 [15] and TRPP2 [17,18] have been detected by immunocytochemistry. In addition, direct evidence of functional ciliary TRPP2-dependent channels has been gained from electrophysiological studies [19,20]. However, there has been no comparable evidence for ciliary TRPM3 channels. While it is known that TRPM3 contributes to the response to hyperosmolality in a cilium-dependent fashion [15], we have seen no stereotypical TRPM3 channels or currents in the cilium, even in the absence of TRPP2. Exogenously expressed TRPM3 has a single-channel conductance of 83 pS when conducting Na^+^ [39], which is similar to the conductance of the ciliary channel. However, expressed TRPM3 channels often show spontaneous activity [39], whereas the ciliary channels are only active with depolarization or micromolar levels of cytoplasmic Ca^2+^ [19,20]. TRPM3 channels conduct Na^+^ and K^+^ equally well [40], while the ciliary channel prefers K^+^ (P_K_/P_Na_ = 2.4 [20] to 7.3 [19]). The permeability of TRPM3 to Ca^2+^ varies between the two splice variants tested. TRPM3α2 is highly permeable to Ca^2+^ (P_Ca_/P_Na_ ≈ 12 [40]), while TRPM3α1 is at least 10 times less conductive to Ca^2+^ [41]. The ciliary channel conducts Ca^2+^ only weakly (P_Ca_/P_Na_ = 0.06 [20]). (We reported a higher Ca^2+^ permeability by linear extrapolation of outward currents to a reversal potential [19]. We are convinced that the inward currents measured directly by Liu et al. [20] give a better estimate of P_Ca_.) The differences between the ciliary channel and TRPM3 must be considered preliminary. In mouse, there are at least 24 splice variants of TRPM3 [25], and the single-channel properties of most have not been described.

The pharmacological profile of the ciliary TRPP2-dependent channel matches that of TRPM3. The open probability of the ciliary channel is strongly increased by pregnenolone sulfate (Figs 1 and 2), as occurs with TRPM3 [35,36]. For exogenously expressed TRPM3α2 channels, pregnenolone sulfate has a half-maximal effect at 23 μM (−80 mV) or 12 μM (+80 mV) [35]. This closely matches the dependence of the ciliary TRPP2-dependent channel on the concentration of pregnenolone sulfate (Fig 2B). The single-channel current-voltage relation of the TRPP2-dependent channel is not significantly changed by addition of pregnenolone sulfate (Fig 1C). It is thus unlikely that pregnenolone sulfate is activating a different class of channel. Furthermore, the incidence of large-conductance channels is not increased by pregnenolone sulfate. In the cilium, pregnenolone sulfate is much less effective from the cytoplasmic face of the membrane, as is true for TRPM3 channels [35,42]. Finally, the ciliary channel is less active in the presence of isosakuranetin, a selective inhibitor of TRPM3 [37] (Fig 3). The combined pharmacological results suggest that TRPM3 may contribute to the TRPP2-dependent channel.

There are three obvious hypotheses surrounding the molecular identity of the large-conductance ciliary channel. A first hypothesis is that it includes TRPP2 subunits but not TRPM3 subunits. This would require that pregnenolone sulfate and isosakuranetin have the same effects on TRPP2 as they have on TRPM3. Even in that case, it would remain to be explained why the ciliary channel requires expression of TRPM3. TRPM3 is not needed for trafficking of TRPP2 to the cilium, as knocking out TRPM3 did not reduce ciliary TRPP2 protein (Fig 5). Nevertheless, no functional large-conductance ciliary channels were detectable in that case. A second hypothesis is that the ciliary channel includes TRPM3 subunits but not TRPP2 subunits. For example, TRPP2 could be required for trafficking of TRPM3 to the cilium. This would account for the pharmacological similarities of the ciliary channel and TRPM3. However, the single-channel conductance and ionic selectivities of the ciliary channel closely resemble those of expressed TRPP2 [20] and, as noted above, do not closely match the known properties of single TRPM3 channels.

The remaining hypothesis is that the ciliary channel may be a heteromultimer that includes both TRPM3 and TRPP2 subunits. It is established that TRPP2 can contribute to heteromultimers in renal epithelial cells. TRPP2 colocalizes with TRPC1 in the cilia of renal cells, and coexpression of the two proteins results in a new type of channel [13]. TRPP2 and TRPV4 colocalize in the cilia of another renal cell line and interact functionally in an expression system [21]. A 23-pS channel has been identified in renal cells (although not in the cilia) that depends on both TRPP2 and TRPV4 [22]. In mIMCD-3 cells, we have not encountered single channels with the reported properties of TRPC1/TRPP2 or TRPV4/TRPP2 heteromultimers. The large-conductance channel we observe depends on expression of both TRPP2 and TRPM3 and is a pharmacological match to TRPM3. Co-immunolocalization or co-immunoprecipitation of the two channel proteins would provide stronger support for the existence of a heteromultimer. However, we were unable to attempt those for lack of a satisfactory antibody against TRPM3. (The antibody we used in a prior study [15] is no longer available to us.) In the future, the existence of a TRPM3/TRPP2 heteromultimer could be better tested by evaluating the effects of mutations to the pore domains of each protein.

Studies using TRPM3-knockout mice have uncovered roles for TRPM3 in nociception [43,44], pupillary light response [45], and retinal function [46]. Complete loss of thermal nociception occurs in TRPM3 knockout mice only with additional knockout of TRPA1 and TRPV1 [44], indicating functional cooperation among these channels. Such cooperativity may occur through heteromerization, although whether such heteromer formation occurs between TRPM3 and other TRP superfamily members is unclear [47]. Nevertheless, despite the renal expression of TRPM3 noted in murine models [15], no renal phenotypes in TRPM3-deficient mice have been reported to date.

Despite expression of TRPM3 in a variety of human tissues including kidney, eye, pancreas, and the central nervous system [39], there is little evidence linking any human disease phenotype to a defect in the *TRPM3* gene. Single nucleotide polymorphisms in the *TRPM3* gene are enriched in individuals with systemic sclerosis [48], aspirin-exacerbated respiratory disease [49], and myalgic encephalomyelitis/chronic fatigue syndrome [50]. Mutation of TRPM3 has been identified in association with an ocular disease phenotype, in which a heterozygous mutation in *TRPM3* segregated with disease in a large (five-generation) pedigree of congenital cataract and glaucoma. There was no mention of any renal phenotypes in these patients [51]. Inactivating mutations in *TRPM3* alone may not be sufficient to cause renal phenotypes including cystic kidney disease, although such mutations could alter/accelerate the progression of polycystic kidney disease in some patients by further disturbing intracellular calcium balance in renal epithelial cells. However, genome-wide association studies of ADPKD patients in whom there is a genotype/phenotype discordance (i.e. more aggressive PKD2-associated disease) that might uncover *TRPM3* as a modifier gene have not been completed.

One can consider whether pregnenolone sulfate might be effective in treating cystic kidney disease. Diseases in this class have several biochemical hallmarks, including lowered cytoplasmic Ca^2+^, elevated intracellular cAMP, cellular hyperproliferation, and excess secretion of fluid [52]. Of these, the reduction in cytoplasmic Ca^2+^ is widely believed to be a root cause of the pathology [52–55]. In cystic cells from human patients and in a rat model, application of a general Ca^2+^ ionophore or Ca^2+^ channel agonists increased cytoplasmic Ca^2+^ and reversed the cystic phenotype [56,57]. Pregnenolone sulfate could be similarly effective, as it likely activates a small ciliary Ca^2+^ influx via the TRPM3/TRPP2-dependent channel. Evidence suggests that TRPP2 (although not necessarily ciliary TRPP2) contributes to normal levels of cytoplasmic Ca^2+^: In renal epithelial cells from TRPP2-knockout mice, cytoplasmic Ca^2+^ is decreased [58]. In autosomal dominant polycystic kidney disease (ADPKD), a minority of patients lack functional TRPP2 [23], and pregnenolone sulfate is not likely to have therapeutic value in the absence of the TRPM3/TRPP2-dependent channel. However, the great majority of ADPKD patients lack functional polycystin-1 (PC1) [59]. In that case, expression of TRPP2 may be preserved, although whether TRPP2 reaches the cilium in the absence of PC1 has been controversial (reviewed by Liu et al. [20]). In autosomal recessive polycystic kidney disease (ARPKD), the primary defect is in fibrocystin/polyductin [60]. In ADPKD or ARPKD patients who retain functional ciliary TRPP2, we speculate that pregnenolone sulfate in the filtrate might promote a beneficial ciliary Ca^2+^ influx. It is not yet known whether this would be sufficient to mitigate the consequences of defects in PC1 or fibrocystin/polyductin. Evaluation of the hypothesis can begin by learning whether pregnenolone sulfate increases cytoplasmic Ca^2+^ in cells lacking PC1 or fibrocystin/polyductin, and whether it can reduce formation of cysts in animal models of polycystic kidney disease that lack mutations in TRPP2. Pregnenolone sulfate is a natural steroid metabolite [61], so a dose that is both effective and non-toxic may be possible.

## Supporting information

S1 Fig Measurement of pregnenolone sulfate concentration in standard external solution. The relation between concentration and absorbance at 215 nm (black line) was established in aqueous samples (gray circles). Concentrations were also measured with pregnenolone sulfate in external solution at nominal concentrations of 50, 200, 300, and 500 μM at times of 10 min (diamonds), 30 min (triangles), or 60 min (open circles). PS, pregnenolone sulfate.
(EPS)

S2 Fig Representative single-channel recordings. Recordings show the activity of single ciliary channels observed while recording in the standard KCl-based internal solution plus 3 μM cytoplasmic free Ca^2+^ and the membrane potentials shown. The concentration of external pregnenolone sulfate (PS) is shown above each set (column). Each set was obtained in one cilium. From many such sets, the open probabilities were averaged and shown in Fig 2B. The dashed lines indicate the current level when the channel was closed. External solutions were chosen as described in the text. For 0 μM and 10 μM pregnenolone sulfate, the standard NaCl-based external solution was used, and the channel current reversed at −67 mV. For 75 μM pregnenolone sulfate, a KCl-based external solution was used, and the channel current reversed near 0 mV.
(EPS)

S3 Fig Quantifying ciliary TRPP2 immunolabeling. Enlarged 3D-rendered views of cilia to illustrate the processing steps used to quantify TRPP2 ciliary labeling. The first row of images shows the overlay of TRPP2 (red) and ARL13B (green) immunolabeling. The second row of images shows just the TRPP2 immunolabeling. The third row shows just the ARL13B immunolabeling. The fourth row shows the volume (gray) that was formed by identifying all the voxels that had an ARL13B intensity value at the defined threshold or greater. The volume image is overlaying the image from the first row. The fifth row of images shows the TRPP2 immunolabeling that was included in the ARL13B-defined volume. We used the median TRPP2 labeling within this volume to represent the TRPP2 labeling for a given cilium. All images in the first and third columns (No Sat.) had contrast enhanced in the same manner with gamma equal to one and no saturated pixels. All the images in the second and fourth columns (C.E.) had the contrast enhanced to allow saturated pixels and, for green, a gamma greater than one (creating a nonlinear relationship between the actual intensity and the displayed intensity) to show both very bright and very dim intensities. No voxels were saturated for the quantification of intensity used to make the graph in Fig 5B. Cilium #1 is an example of cilia with long axes that project well above the cell and are easily isolated from the cytoplasmic TRPP2 labeling. Cilium #4 is an example of cilia with long axes parallel to the surface of the monolayer that are less easily isolated from the cytoplasmic TRPP2 labeling. The same threshold of ARL13B intensity, which was used to define the ciliary volume, was used for all images used in the quantification. This threshold was a compromise value that missed a few very lightly ARL13B-labeled ciliary parts in order to avoid making the volume on more ARL13B-brightly labeled cilia (cilium #2) so large that substantial cytoplasmic TRPP2 labeling would be included. In the image that shows cilium #1, row 3, note the faint green spherical structure in the cytoplasm (arrow); rarely, one of these spheres was bright enough to be thresholded and was then excluded manually. XYZ scale bars = 1 μm.
(JPG)

S1 Data A spreadsheet listing the raw data underlying the reported results. (XLSX)

S1 Table Best-fitting Boltzmann functions relating open probability and voltage. For each of four concentrations of external pregnenolone sulfate, the relation between open probability and voltage (Fig 2B) was fit to a Boltzmann function as described previously [19]. The concentration of cytoplasmic Ca^2+^ was 3 μM. For each function, the Boltzmann constants *V*_1/2_ and *k* are shown, as well as the strength (R^2^) and significance (*P*) of the fit. *V*_*m*_, membrane potential; *V*_1/2_, the potential at which open probability is 0.5; *k*, a slope factor; PS, pregnenolone sulfate. Pregnenolone sulfate at concentrations of 1 μM to 75 μM significantly shifted *V*_1/2_ to more negative values (one-way ANOVA on *V*_1/2_ with Holm-Sidak all pairwise comparison; *P* < 0.001 for all comparisons). The data shown in Fig 2A (0.1 μM free Ca^2+^) were insufficient to define Boltzmann functions.
(PDF)
