# Supplementary figures and images for: The TRPP2-dependent channel of renal primary cilia also requires TRPM3
Source: PLoS One. 2019 Mar 18;14(3):e0214053. doi: 10.1371/journal.pone.0214053 (PMC6422334; doi:10.1371/journal.pone.0214053)

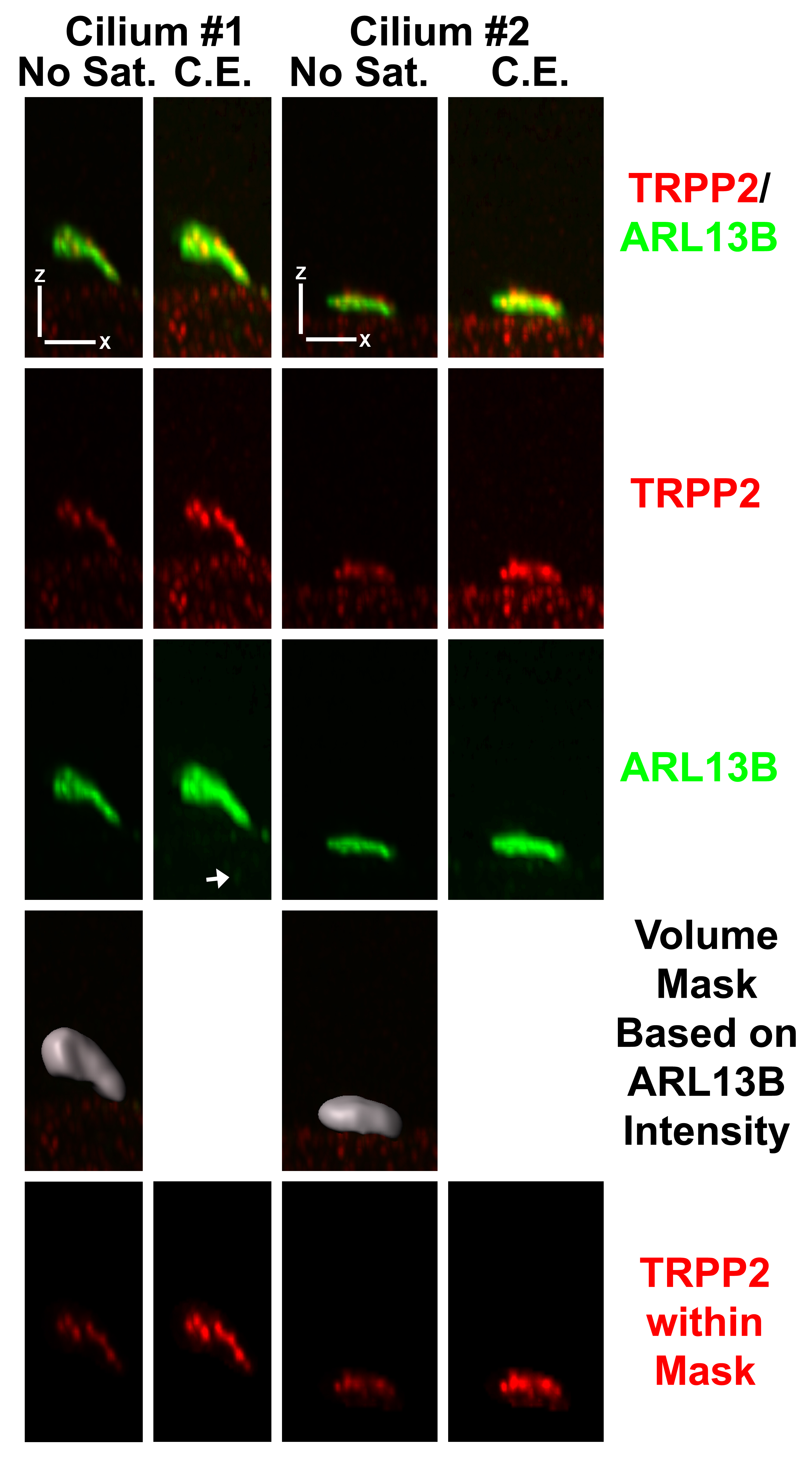

Supplement: S3 Fig — Enlarged 3D-rendered views of cilia to illustrate the processing steps used to quantify TRPP2 ciliary labeling. The first row of images shows the overlay of TRPP2 (red) and ARL13B (green) immunolabeling. The second row of images shows just the TRPP2 immunolabeling. The third row shows just the ARL13B immunolabeling. The fourth row shows the volume (gray) that was formed by identifying all the voxels that had an ARL13B intensity value at the defined threshold or greater. The volume image is overlaying the image from the first row. The fifth row of images shows the TRPP2 immunolabeling that was included in the ARL13B-defined volume. We used the median TRPP2 labeling within this volume to represent the TRPP2 labeling for a given cilium. All images in the first and third columns (No Sat.) had contrast enhanced in the same manner with gamma equal to one and no saturated pixels. All the images in the second and fourth columns (C.E.) had the contrast enhanced to allow saturated pixels and, for green, a gamma greater than one (creating a nonlinear relationship between the actual intensity and the displayed intensity) to show both very bright and very dim intensities. No voxels were saturated for the quantification of intensity used to make the graph in Fig 5B. Cilium #1 is an example of cilia with long axes that project well above the cell and are easily isolated from the cytoplasmic TRPP2 labeling. Cilium #4 is an example of cilia with long axes parallel to the surface of the monolayer that are less easily isolated from the cytoplasmic TRPP2 labeling. The same threshold of ARL13B intensity, which was used to define the ciliary volume, was used for all images used in the quantification. This threshold was a compromise value that missed a few very lightly ARL13B-labeled ciliary parts in order to avoid making the volume on more ARL13B-brightly labeled cilia (cilium #2) so large that substantial cytoplasmic TRPP2 labeling would be included. In the image that shows cilium #1, r [file pone.0214053.s003.jpg]
