# Supplementary material for: The TRPP2-dependent channel of renal primary cilia also requires TRPM3
Source: PLoS One. 2019 Mar 18;14(3):e0214053. doi: 10.1371/journal.pone.0214053 (PMC6422334; doi:10.1371/journal.pone.0214053)
Supplement: S1 Table — For each of four concentrations of external pregnenolone sulfate, the relation between open probability and voltage (Fig 2B) was fit to a Boltzmann function as described previously [19]. The concentration of cytoplasmic Ca2+ was 3 μM. For each function, the Boltzmann constants V1/2 and k are shown, as well as the strength (R2) and significance (P) of the fit. Vm, membrane potential; V1/2, the potential at which open probability is 0.5; k, a slope factor; PS, pregnenolone sulfate. Pregnenolone sulfate at concentrations of 1 μM to 75 μM significantly shifted V1/2 to more negative values (one-way ANOVA on V1/2 with Holm-Sidak all pairwise comparison; P < 0.001 for all comparisons). The data shown in Fig 2A (0.1 μM free Ca2+) were insufficient to define Boltzmann functions. (PDF) [file pone.0214053.s005.pdf]

[PS], μM	V_m (mV) tested	n	Boltzmann constants		R^2
			$V_{1/2}$ (mV)	k (mV)	
0	−40 to +60	6-9	7.6 ± 0.9	13.2 ± 0.8	0.993
1	−40 to +60	7-10	−5.6 ± 1.1	13.5 ± 1.0	0.990
10	−40 to +50	9-11	−21.4 ± 1.4	18.3 ± 1.5	0.979
75	−140 to −20	4-9	−67.2 ± 1.0	14.8 ± 0.9	0.992
